# The Impact of Physical Activity on the Outcomes of Active Surveillance in Prostate Cancer Patients: A Scoping Review

**DOI:** 10.3390/cancers16030630

**Published:** 2024-02-01

**Authors:** Aldo Brassetti, Loris Cacciatore, Alfredo Maria Bove, Umberto Anceschi, Flavia Proietti, Leonardo Misuraca, Gabriele Tuderti, Rocco Simone Flammia, Riccardo Mastroianni, Maria Consiglia Ferriero, Giuseppe Chiacchio, Simone D’Annunzio, Rigoberto Pallares-Mendez, Riccardo Lombardo, Costantino Leonardo, Cosimo De Nunzio, Giuseppe Simone

**Affiliations:** 1 IRCCS “Regina Elena” National Cancer Institute, Department of Urology, Via Elio Chianesi 53, 00144 Rome, Italy; aldo.brassetti@ifo.it (A.B.); alfredo.bove@ifo.it (A.M.B.); umberto.anceschi@gmail.com (U.A.); flavia.proietti@ifo.it (F.P.); leonardo.misuraca@gmail.com (L.M.); gabriele.tuderti@gmail.com (G.T.); roccosimone92@gmail.com (R.S.F.); riccardo.mastroianni@ifo.it (R.M.); maria.ferriero@ifo.it (M.C.F.); gipeppo1@gmail.com (G.C.); simone.dannunzio@ifo.it (S.D.); rigo_pallares@hotmail.com (R.P.-M.); costantino.leonardo@ifo.it (C.L.); puldet@gmail.com (G.S.); 2 “Sapienza” University of Rome, Department of Urology, Via di Grottarossa 1035, 00189 Rome, Italy; rlombardo@me.com (R.L.); cosimo.denunzio@uniroma1.it (C.D.N.)

**Keywords:** prostate cancer, active surveillance, physical activity, exercise, lifestyle, disease progression

## Abstract

**Simple Summary:**

The present review explores the impact of physical activity (PA) on patients with prostate cancer (PCa) managed through active surveillance (AS). Specifically, the correlation between the duration, frequency, and intensity of physical exercise and the risk of tumor reclassification has been investigated. We pointed out inconsistencies in PA assessment and conflicting findings regarding its association with AS outcomes. Nevertheless, several studies suggest that active individuals may experience reduced risk of PCa progression. This renders PA a cost-effective approach for minimizing the need for definitive treatment, thereby classifying it within tertiary prevention strategies. Given the increasing number of patients diagnosed with PCa, this strategy may assume significant relevance for the public health in the coming years.

**Abstract:**

Introduction: Active surveillance has emerged as a valid therapeutic option in patients with low-risk prostate cancer, allowing for the deferral of definitive treatment until the time of possible disease progression. Although it is known that physical activity plays a protective role in the onset and progression of this tumor, its impact on patients with low-risk disease who are managed with active surveillance remains unclear. Our scoping review aims to summarize the existing evidence on this subject. Evidence Acquisition: On 9 April 2023, a systematic search was conducted using the PubMed and Scopus databases. The search employed the combination of the following terms: (“prostate cancer” OR “prostate tumor”) AND (“active surveillance”) AND (“physical activity” OR “physical exercise” OR “physical intensive activity” OR “intensive exercise”) AND (“lifestyle”). Out of the 506 identified articles, 9 were used for the present scoping review, and their results were reported according to the PRISMA-ScR statement. Evidence Synthesis: We discovered a lack of uniformity in the assessment of PA and its stratification by intensity. There was no consensus regarding what constitutes cancer progression in patients choosing expectant management. In terms of the impact of PA on AS outcomes, conflicting results were reported: some authors found no correlation, while others (six of total studies included) revealed that active men experience smaller increases in PSA levels compared to their sedentary counterparts. Additionally, higher levels of exercise were associated with a significantly reduced risk of PCa reclassification. Conclusion: Due to the heterogeneity of the methodologies used in the available studies and the conflicting results reported, it is not possible to draw definitive conclusions concerning the role physical activity may play in the risk of prostate cancer progression in men managed with active surveillance.

## 1. Introduction

Prostate cancer (PCa) is a significant global healthcare concern, with an estimated 288,300 new cases reported in the United States in 2023 [1]. In the 1990s, we witnessed a sudden increase in the incidence of this disease, following the introduction of prostate-specific antigen (PSA)-based screening protocols. Though, as a consequence of the warning from the United States Preventive Services Task Force [2], a subsequent reversal of this trend was observed [3,4]. Early diagnosis has certainly resulted in a significant reduction in mortality (−53% compared to 1993), while also leading to the identification of a large number of indolent neoplasms [5,6]. According to a recent observational study that was conducted on 82,429 males who adhered to a PSA-based screening program, in the United Kingdom, the overall incidence of PCa is low (3%; *n* = 2664). Out of the 1643 patients who agreed to be randomly assigned to active monitoring, surgery, or radiotherapy, two-thirds were found to have a low-risk disease. At a median follow-up of 15 years, cancer-specific mortality (CSM) was low, regardless of the treatment received (3.1% vs. 2.2% vs. 2.9%; *p* = 0.53). Throughout the study period, a total of 356 deaths were recorded, resulting in a 22% all-cause mortality rate, but again, there was no significant difference between the three study arms [6].

Therefore, considering the significant morbidity associated with definitive treatment modalities [7], most patients with low-risk cancers are now commonly managed conservatively [8]. This paradigm involves regular monitoring with an intent to proceed to either surgery or radiation therapy in case of disease reclassification [8], which is not uncommon, [9] as it occurs in one quarter of cases within 3 years and in half of the patients within 5 years from diagnosis [10,11].

There is solid evidence indicating that physical activity (PA) has an impact on tumor development and progression. Specifically, increased levels of exercise have not only been linked to an improved quality of life for PCa patients, but also to a decreased risk of tumor diagnosis, recurrence, and CSM [12,13,14]. Substantiating this assertion, a recent systematic review by Nader et al. underscores the substantial impact of aerobic and endurance training on enhancing the quality of life for individuals diagnosed with PCa, supporting their integration into the broader spectrum of patient care [15]. However, the role of exercise in the context of active surveillance (AS) remains unclear, and its effectiveness in preventing disease reclassification is still a matter of debate within the urological community.

Our review aims to provide a comprehensive and organized summary of existing evidence regarding how the frequency, duration, and intensity of PA impact the risk of tumor progression in men undergoing AS.

## 2. Evidence Acquisition

To clarify whether PA plays a role in reducing the risk of reclassification in patients with PCa managed with AS, we have conceived this scoping review [16]. The PICO framework [17] was employed to facilitate a comprehensive search process with a focus on the following specific parameters:

- Population: men of all ages affected by low-risk prostate cancer.
- Intervention: active surveillance.
- Comparison: physical activity levels.
- Outcome: tumor progression/disease reclassification.

On 9 April 2023, a literature search was conducted using the PubMed and Scopus databases to identify studies that met the predetermined inclusion criteria: no chronological restriction was applied. The query utilized the following keywords in combination: (“prostate cancer” OR “prostate tumor”) AND (“active surveillance”) AND (“physical activity” OR “physical exercise” OR “physical intensive activity” OR “intensive exercise”) AND (“lifestyle”).

Only English-language papers were considered for inclusion; animal studies, case reports, conference abstracts, letters to the editor, editorials, and reviews were excluded. To ensure rigorous selection and obtain consistent and comparable data, we also excluded studies that did not clearly define:

- the criteria for inclusion in AS protocols
- the definition of “disease progression”
- the methods used to assess and quantify PA
- interventions conducted by researchers on patients’ PA.

The minimum duration of these interventions was set at 6 months, allowing sufficient time to observe and measure any potential effect produced.

Using the Covidence Systematic Review Management^®^ (Veritas Health Innovation, Melbourne, Australia), two independent authors (L.C. and A.B.) conducted a thorough screening of all retrieved records. In cases of discrepancies, a discussion was held to reach a resolution. The full texts of the screened papers were further assessed for eligibility: those remaining were included in the present review. The three processes of identification, screening, and selection of scientific articles to be included in our review were supervised by an experienced author (G.S.).

The results of our search were reported according to the PRISMA (Preferred Reporting Items for Systematic Reviews and Meta-Analyses) statement checklist, and its dedicated extension for scoping reviews (PRISMA-ScR) [18,19]. We registered our scoping review on OSF registries (https://doi.org/10.17605/OSF.IO/GB4HK, accessed on 21 November 2023).

## 3. Results of the Search

**Figure 1:**
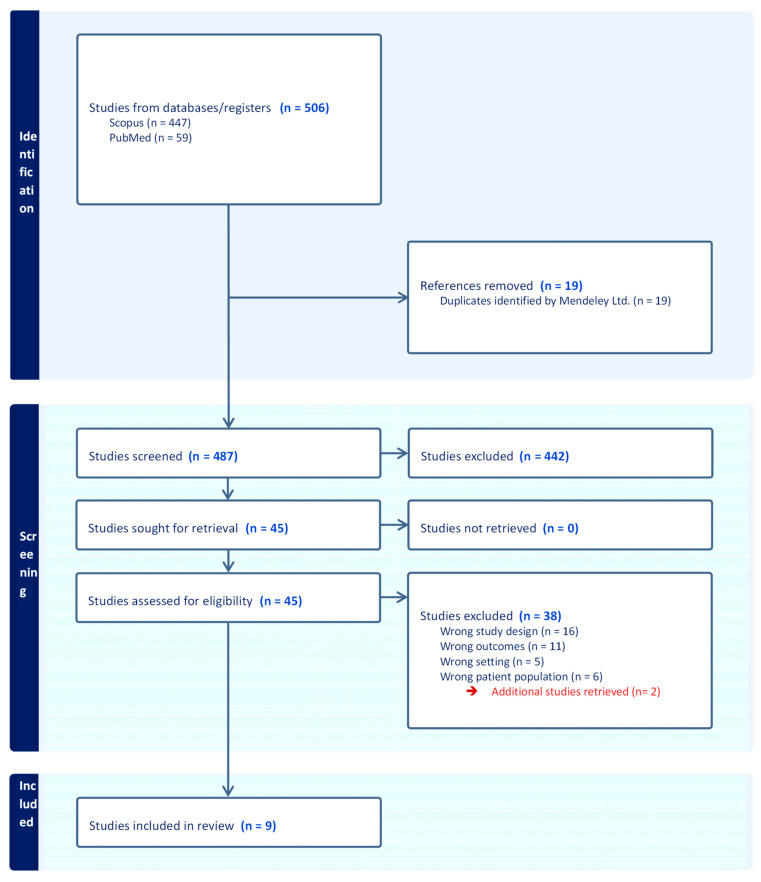
Flow diagram of the selection process regarding PCa progression and physical activity (PRISMA guidelines).

The literature search yielded 506 papers, comprising Scopus (*n* = 447) and PubMed (*n* = 59). Through an automatic process, 19 duplicate studies were excluded. After screening titles and abstracts of the remaining 487 references, we additionally discarded 442 records, which were not pertinent to the objective of the study. Thereafter, we assessed the full texts of the remaining 45 studies for eligibility, and 7 were accepted. Afterwards, the reference list of qualifying papers was reviewed, and we identified 2 additional papers not initially captured in the first selection. Finally, we included a total of 9 studies, of which 2 were prospective studies, 4 were retrospective studies, and 3 were randomized controlled trials (RCTs) (Table 1). Figure 1 (flow diagram) provides a graphical representation of the literature search and screening process.

## 4. Evidence Synthesis

Overall, the results discussed in this review are based on data obtained from a total of 5140 patients with PCa included in 9 studies (Table 1). Despite strict paper selection, we observed considerable heterogeneity among those comprised in our review, which is already evident from the inclusion criteria for active surveillance (AS). In fact, while most authors offered this treatment strategy exclusively to patients diagnosed with low-risk PCa [28,29,30,31], some expanded the indication to include men with low-volume Gleason 7 (3 + 4) diseases [32,33,34].

Various methods were used for measuring PA: the predominant approach was to employ validated questionnaires, such as the Global Physical Activity Questionnaire (GPAQ) [26], the Godin Leisure-Time Exercise questionnaire (GLTEQ) [27], and the Physical Activity Scale for the Elderly (PASE) [28]. On the contrary, Eriksen et al. [24] provided each patient with an accelerometer. In most studies, the Metabolic Equivalent of Task (MET) was used as a unit of measure to report exercise intensity, as well as its duration and frequency [22,23,25,27]: three studies used the cut-off of MET ≥ 6 to define “intense” physical activity [22,23,25].

Moreover, while some authors explored the correlation between PA and the risk of tumor reclassification at biopsy (defined as up-grading and/or up-staging of PCa) [25,27,28], others assessed the impact of exercise on the increase in PSA values [20,21,22,23,24], on the need for unplanned diagnostic investigations (such as additional prostate biopsies or magnetic resonances), and on the resort to definitive treatment [21,22].

## 5. Discussion

All currently available treatments for PCa carry a significant risk of various adverse effects, including impotence, incontinence, infections, chronic inflammation of pelvic organs, and, ultimately, radiation-induced carcinogenesis. Such significant toxicity greatly affects patients’ overall well-being [7]. For these reasons, since approximately two out of every three newly diagnosed malignancies are classified as low-grade, increasing numbers of men are being offered AS. This approach involves regular monitoring of the disease, and curative treatments are initiated only if cancer shows signs of progression [8]. Within 15 years from diagnosis, up to 60% of men under active monitoring undergo definitive treatment [6,35]. Consequently, there is significant scientific interest in identifying cost-effective and safe interventions that may potentially delay or eliminate the need for surgery and radiation.

Nowadays, there is a growing body of evidence suggesting that men’s lifestyle has a significant impact on PCa incidence and aggressiveness. Several studies in the literature have found that higher levels of PA are associated with a reduced risk of tumor diagnosis, recurrence, and death [14,36,37]. Specifically, observational studies have consistently shown that vigorous exercise after cancer diagnosis leads to more favorable oncological outcomes [29,38,39,40]. While a recent systematic review conducted by Dovey et al. revealed a positive influence of PA on mental health for PCa patients, with 10 out of 15 articles supporting this connection; additionally, concerning oncological outcomes, 26 out of 44 studies demonstrated a positive impact, especially for moderate to vigorous PA, further emphasizing the potential benefits of exercise in the context of PCa [41]. Consequently, it has been incorporated into the ASCO Clinical Practice Guidelines for Prostate Cancer [42]. However, the role of PA in the context of AS is still a subject of debate within the urological community.

While the exact mechanisms underlying the protective effects of exercise are still not fully understood, it has been reported that various pathways may contribute to this effect [14]. One key mechanism through which physical exercise exerts its influence is by modulating of gene expression. The GEMINAL study demonstrated that, following three months of dietary changes and daily brisk walking, telomere length increased [43]. This intervention also impacted the expression of genes involved in protein intracellular transportation, metabolism, and phosphorylation [44]. More specifically, vigorous PA is capable of boosting telomere elongation and modulating gene expression, upregulating cell cycling and DNA repair pathways, and modulating the pathways of Nrf2-mediated oxidative stress response [43,45]. Similarly, Magbanua et al. found that engaging in high-intensity training for at least three hours per week enhanced the expression of specific antioncogenes in prostate tissues [45]. Furthermore, increased levels of PA have been shown to improve insulin resistance and interact with the levels of various circulating tumor-promoting proteins, such as insulin-like growth factor-1 (IGF-1), which has both mitogenic and anti-apoptotic effects. In fact, the apoptotic rate significantly increased in lymph node carcinoma of the prostate (LNCaP) cells incubated with serum obtained from daily trained low-risk PCa patients [20]. Additionally, other pathways have been identified (IGF-3, p53, p21, caspases, and Bcl-2) and hypothesized to contribute to the protective effects of PA, including reduced oxidative stress, inflammation, and enhanced immune surveillance [14,46].

In recent years, the landscape of therapeutic options for PCa has evolved significantly with the introduction and widespread adoption of focal therapies (namely cryotherapy ablation, focal photodynamic therapy, and high-intensity focused ultrasound). These new treatments have been added to established modalities such as radical prostatectomy, radiation therapy, and hormone-deprivation therapy. Technological advancements have played a crucial role in making PCa treatment less invasive, prioritizing oncological safety while reducing toxicity. The availability of a broad range of treatments reflects the urological community’s interest in a holistic approach to PCa. It takes into account both oncological efficacy and the preservation of quality of life.

Given the substantial morbidity associated with definitive treatment options, many low-risk PCa patients are now choosing conservative management strategies [7,8]. In 2016, AS became the standard of care for managing low-risk PCa patients in the United States. Community-based registries have reported a notable increase in its utilization, ranging from 40% to 50% in the current decade [47]. This represents a substantial rise compared to historical rates, which rarely exceeded 10% [8,48]. A growing body of evidence suggests that surveillance maintains a good quality of life while posing minimal oncologic risks in the short to intermediate term [49].

The purpose of this deferred-treatment strategy is to avoid surgery or radiotherapy-related morbidity in men with a minimally aggressive localized disease and a life expectancy of 10 years or more. These individuals do not require immediate treatment but rather need to determine the optimal timing for treatment when necessary. Therefore, according to international guidelines, men on AS should undergo comprehensive monitoring through structured programs that include regular follow-up visits with PSA testing, clinical examinations, multiparametric magnetic resonance imaging, and repeated prostate biopsies. The decision to undergo definitive treatment is based on predefined thresholds that indicate the potential emergence of life-threatening disease [32]. It is not uncommon for tumors to be reclassified during surveillance [9], with one quarter of cases experiencing reclassification within 3 years and half of the patients within 5 years from diagnosis [10,11]. The deferred-treatment strategies, as said, allow for the use of definitive therapies only in the case of disease progression, while check-ups are scheduled to ensure that surgery or radiotherapy is administered when the tumor is still curable. This approach helps alleviate the burden that an oncological diagnosis could have on the quality of life of low-risk PCa patients and simultaneously represents a cost-effective treatment option for healthcare systems [50]. Consequently, there is a growing interest in identifying modifiable factors that can influence disease progression [51], providing valuable forms of tertiary prevention [52].

Given the well-established simultaneous roles of race, genetic background, and sexual behavior in the development and progression of PCa, dietary and lifestyle factors are gaining recognition as significant determinants of carcinogenesis. For instance, it has been emphasized that consuming a generous amount of vegetables, particularly those rich in lycopene, selenium, vitamin E, and vitamin C, is associated with a reduced risk of developing this disease [30,33]. Moreover, epidemiological and migration studies have unveiled that individuals who prefer a low-fat, primarily plant-based diet demonstrate a lower incidence of clinically significant PCa [31,53]. Engaging in regular physical exercise, which helps reduce adiposity while lowering blood levels of inflammatory cytokines, has also emerged as a protective factor against the risk of PCa diagnosis and aggressiveness at prostate biopsy [36]. Other studies have indicated that an active lifestyle also reduces the risk of tumor recurrence and progression after primary treatment [34,54]. Therefore, the inclusion of physical exercise into the American Society of Clinical Oncology (ASCO) Clinical Practice Guidelines on PCa [42] signifies a strategic recognition of its crucial role in influencing the natural history of this tumor.

Based on our review, several considerations can be done. First, the criteria used by the authors to identify patients eligible for AS have not been consistent across the nine different studies. While in most cases, surveillance has been offered to patients with low-risk disease, in three cases, the indication has been extended to individuals with low-volume Gleason 7 (3 + 4) PCa [42,43,47]. In fact, a recent meta-analysis has shown significantly worse oncological results in patients with intermediate-risk PCa who were offered AS compared to those with low-risk disease. However, in a subgroup analysis comparing outcomes of patients with ISUP < 2 (*n* = 1900) and intermediate- and low-risk PCa, no statistically significant difference was found in terms of treatment-free survival or risk of developing metastases (RR: 1.03, 95% CI: 0.62–1.71 and RR: 2.09, 95% CI: 0.75–5.82, respectively) [10.1016/j.clgc.2020.05.008] [10.1016/j.euo.2022.07.004].

Moreover, we did not find any uniformity in the evaluation of PA and its stratification by intensity. The most rigorous approach was indisputably that of Eriksen et al., who chose to monitor patients using wearable devices equipped with accelerometers and gyroscopes [55]. This allowed the researchers to accurately and reliably record the frequency and duration of physical exercise performed by the men enrolled in the study, while also estimating its intensity. On the other hand, other authors resorted to validated questionnaires, which do not allow for precise recording of PA but require the patient to self-estimate the average physical effort over a certain period of time. In our recently published multicenter study [28], we used the PASE (Physical Activity Scale for the Elderly) to differentiate sedentary patients (PASE ≤ 65) from moderately active (65 < PASE < 125) and active (PASE ≥ 125) ones. Also Guy et al. [26] and Papadoupoulos et al. [27] used surveys to objectively assess the level of PA of their patients, resorting to the Global Physical Activity (GPAQ) and Godin Leisure-Time Exercise (GLTEQ) questionnaires, respectively. More in detail, the latter was used to calculate the metabolic equivalent minutes per week (MET-min/wk) in order to stratify patients into inactive (<210 MET-min/wk), insufficiently active (210 < MET-min/wk < 500), active (500 < METmin/wk < 1000), or highly active (>1000 MET-min/wk). Also Richman et al. [22], Kenfield et al. [23], and Vandersluis et al. [25] used metabolic equivalents to quantify PA, but the chosen unit of measurement was different (MET-hour/wk), as well as the cut-off value to define high-intensity exercises (≥6 MET-hour/wk). It is interesting to observe the tendency of most authors to refer to the Metabolic Equivalent of Task (MET) as a unit of measurement to report PA intensity. The MET is a derived unit, defined as a multiple of the resting metabolic rate (RMR), which in turn represents the energy expenditure of a reference individual while quietly seated. The RMR can be measured by absolute gas exchange, absolute thermal output, or steady-state diet in a sedentary state.

Regardless of the methods used to measure its intensity, most authors agree on the need to define the minimum duration and frequency of PA eventually capable of reducing the risk of PCa progression. Kenfield et al. [23] observed that at least 3 h of intense exercise per week are required to obtain beneficial effects from an oncological perspective, while Eriksen et al. [24] considered at least 45 min of training for 3 days per week or 10,000 steps per day for 6 months to be necessary. Frattaroli and Ornish, on the other hand, recommend 30 min of walking per day for 6 days per week [20,21].

Based on our review, the various authors did not find a consistent outcome that would confirm the potential protective effect of physical activity in patients under active surveillance. In fact, some colleagues focused on the effect of exercise on the risk of suspicious PSA elevations [22,23,24], while others investigated its impact on clinical progression at multiparametric magnetic resonance imaging and/or digital rectal examination [20,21]. There were also studies that examined the relationship between PA and tumor upgrading at prostate biopsy [25,26,27,28]. Only one study assessed the impact of exercise on the risk of CSM [23].

Finally, the clinical studies included in this review have yielded conflicting results. Some authors have found no correlation between engaging in physical exercise and the risk of tumor progression [24,25,27]. Specifically, two RCTs failed to demonstrate a link between PA and PSA kinetics [21,24]. However, after observing the participants of the Prostate Cancer Lifestyle Trial for two years, it was noted that the rate of definitive treatment was significantly lower among active men compared to the control group (5% vs. 27%, *p* = 0.005) [21]. On the contrary, other studies suggested that physical exercise may indeed have an impact on the progression of the disease [20,22,23,26]. For example, Ornish et al. conducted a study that revealed active men experienced smaller increases in PSA levels compared to their sedentary counterparts [20]. Moreover, our recent research displays evidence that higher levels of exercise are associated with a significantly reduced risk of PCa reclassification (OR: 0.98; 95% CI: 0.97–0.99; *p* = 0.02) [28]. Similar findings were reported by Guy [26] and Richman [22]. In a retrospective study based on data from 1455 American males, Richman et al. demonstrated that individuals who engage in at least three hours of brisk walking per week experience a significant decrease in the risk of tumor reclassification (HR: 0.43; 95% CI: 0.21–0.91; *p* = 0.03). Notably, the protective effect of PA persists even in those who engage in mild exercise (HR: 0.37; 95% CI: 0.11–1.22; *p* = 0.10). Furthermore, the frequency of steps taken during walks, rather than the duration, was found to be statistically associated with a reduction in the risk of PCa progression (HR: 0.52; 95% CI: 0.29–0.91; *p* = 0.01) [22]. Lastly, some of the studies included in our review have documented the protective effect of PA on certain endocrine disorders such as obesity, hypercholesterolemia, and metabolic syndrome. These pathological conditions have already been extensively investigated as possible causes of the onset and progression of PCa, albeit with conflicting results [36,37,55,56]. More specifically, our previous findings concluded that MetS is associated with an increased risk of high-grade cancers at prostate biopsy, adverse features at final pathology, disease recurrence, and CSM [37,55,56]. Therefore, the findings reported by Ornish [20], Eriksen [24], and Frattaroli [21] can provide a valid starting point for physio-pathological speculations regarding the correlation between PA and the natural history of PCa.

Our conclusions must be considered in light of the limitations of the studies included in the present review. As mentioned above, there is no consensus on the definition of PA, and the methodologies employed to measure it vary significantly. Most studies rely on validated questionnaires, which offer a retrospective, approximate, and subjective quantification of the patients’ PA, without distinguishing between sports and other types of exercise. Only one study utilized wearable devices to objectively monitor and document the daily energy expenditures of the participants. Another limitation of the analyzed studies lies in the inconsistent definitions of the oncological outcomes examined. Not all cases of progression during AS were histologically confirmed, and some authors considered elevated prostate-specific antigen (PSA) levels or radiological evidence of worsening index lesions as indicators of increased cancer aggressiveness.

Despite these limitations, our study offers valuable insights into the existing body of literature regarding the relationship between PA and disease progression in patients undergoing AS for PCa. By summarizing the available evidence, we presented a comprehensive overview of the methodologies used to assess physical exercise duration, frequency, and intensity. We also reported the different oncological outcomes assessed across the studies. This critical examination highlights the need for standardized approaches in future research. To accurately assess the impact of exercise on the risk of PCa progression in patients managed with AS, further randomized clinical trials with extended follow-up periods, incorporating the use of wearable devices for daily PA measurement, are warranted.

## 6. Conclusions

Given the high prevalence of PCa and the significant proportion of cases classified as low-risk diseases, taking into account the substantial toxicity associated with definitive treatments, AS is considered a viable option. However, it is crucial to acknowledge that the risk of tumor progression for these individuals cannot be disregarded, and all possible measures should be taken to minimize it. Although the available evidence does not permit definitive conclusions regarding the effectiveness of PA in reducing this risk, this review underscored the well-established positive impact of exercise on the overall health of cancer patients, highlighting positive association between higher levels of PA and reduced risk of PCa reclassification.

In light of these findings, we emphasize the importance of continued investigation in this area to provide more definitive insights. To achieve this goal, it is imperative to conduct clinical trials that involve individuals who meet inclusion criteria endorsed by the international scientific community. The PA of these individuals (including activities at home, work, and sports) could be continuously monitored using wearable devices for a minimum duration of 6 months, and findings should be reported using consistent units of measurement.

## Figures and Tables

**Table 1:** Included studies arranged by type of study, number of patients, population and intervention details, assessment of physical activity, stratification by intensity, measured oncological outcomes, association PA and progression of PCa, and quantitative results.

Reference	Study Design	Number of Pts	Population Details	Intervention Details	Assessment of Physical Activity	Stratification by Intensity (Score—Scale)	Measured Oncological Outcomes	Association PA and Progression of PCa (Yes/No)	Quantitative Results
Ornish et al., 2005 [20]	Randomized controlled trial	93	GS < 7 PSA 4–10 ng/mL T1-T2 stage	Experimental group vs. control group	Self-reporting questionnaire (0: no adherence; 1: adherence)	Not reported	Increased PSA, LNCaP cell growth	yes	(r = −0.23, *p* < 0.035)
Frattaroli et al., 2008 [21]	Randomized controlled trial	93	GS < 7 PSA 4–10 ng/mL	Experimental group vs. usual care	Self-reporting questionnaire (0%: no adherence; 100%: adherence)	Not reported	Conventional PCa treatment, increased PSA	yes/no	r = 0.255; CI: (0.053–0.437); *p* < 0.005/*p* > 0.05 (PSA)
Richman et al., 2011 [22]	Retrospective study (Cross-sectional study)	1455	Clinically localized prostate cancer	Individual physical activity	Metabolic equivalent task (MET-hour/week) value	Non vigorous PA: MET < 6 Vigorous PA: MET ≥ 6	Increased suspicious PSA, secondary treatment	yes	HR 0.43; 95%CI: (0.21–0.91); *p* = 0.03
Kenfield et al. 2011 [23]	Prospective study	2705	GS < 7 T1-T2 stage	Different type of physical activity	Metabolic equivalent task (MET-hour/week) value	Non vigorous PA: MET < 6 Vigorous PA: MET ≥ 6	Increased suspicious PSA, PCa specific death	yes	HR 0.44; 95% CI: (0.17–1.15)
Eriksen et al., 2012 [24]	Randomized controlled trial	26	Clinically localized prostate cancer	Experimental group vs. control group	Accelerometers/Gyroscopes	Not reported	Increased suspicious PSA	no	HR 0.2; 95% CI: (−2.1–2.6)
Vandersluis et al., 2016 [25]	Retrospective study	131	GS ≤ 7 PSA < 10 ng/mL T1c-T2a stage	Individual physical activity	Metabolic equivalent task (MET-hour/week) value	Non vigorous PA: MET < 6 Vigorous PA: MET ≥ 6	Tumor upgrading at biopsy	no	95% CI: (0.77–1.16); *p* = 0.29/ 95% CI: (0.55–1.02); *p* = 0.066
Guy et al., 2019 [26]	Retrospective study (Cross-sectional study)	131	GS ≤ 7 PSA < 10 ng/mL T1c-T2a stage	Recreational and total Physical activity	Global Physical Activity Questionnaire (GPAQ)/ Adjusted MET value for activity = Standard MET value for activity × ([3.5 mL O_2_/kg·min]/[3.6145 − (0.0367 × BMI) − (0.0038 × age) + (0.1790 × 2)])	Moderate intensity: small increases in Breathing and heart rate Vigorous intensity: large increases in breathing and heart rate	Tumor Tumor upgrading at biopsy	yes	OR 0.42; 95% CI: (0.20–0.85) (p-trend = 0.027)
Papadopoulos et al., 2019 [27]	Retrospective study (Cross-sectional study)	421	GS ≤ 6 PSA < 10 ng/mL Stage ≤ T2a	Individual physical activity	Godin Leisure-Time Exercise Questionnaire (GLTEQ)/ Metabolic equivalent task (MET-hour/week) value	Inactive: <210 MET-min/wk) Insufficiently active: 210 < MET-min/wk < 500 Active: 500 < MET-min/wk < 1000 Highly active: > 1000 MET-min/wk	Tumor upgrading at biopsy	no	HR 1.11; 95% CI: (1.03–1.21)
Brassetti et al., 2021 [28]	Retrospective study (Cross-sectional study)	85	ISUP 1 ≤ 2 positive cores, stage T1c-T2a, PSA ≤ 10 ng/mL, PSA density < 0.2	Individual physical activity	Physical Activity Scale for the Elderly (PASE)	Sedentary: PASE ≤ 65 Moderately active: 65 < PASE < 125 Active: PASE ≥ 125	Tumor upgrading at biopsy	yes	HR: 0.987; 95%CI: (0.977–0.998); *p* = 0.016
